# EEG correlates of impaired anticipation processes in the early stages of schizophrenia

**DOI:** 10.1192/j.eurpsy.2022.804

**Published:** 2022-09-01

**Authors:** M. Slavutskaya, I. Lebedeva, M. Omelchenko, E. Abdullina, S. Karelin

**Affiliations:** 1 Mental Health Research Center, Laboratory Of Neuroimaging And Multimodal Analysis, Moscow, Russian Federation; 2 Lomonosov Moscow State University, Department Of The Highest Nervous Activity, Moscow, Russian Federation; 3 Mental Health Research Center, Department Of Youth Psychiatry, Moscow, Russian Federation

**Keywords:** go/no-go delay paradigm, anticipation, saccade, slow waves

## Abstract

**Introduction:**

An impairment of anticipation processes is considered as a common deficiency in schizophrenia (Kveraga et al., 2007), however its neural mechanisms remain poorly understood.

**Objectives:**

The aim of the study was to analyze CNV-like slow negative waves during the pre-target stimuli waiting period in patients with the first episode of the disease.

**Methods:**

32-channels EEGs during “Go / No go delay” saccadic paradigm have been recorded in 16 young male patients with illness duration less than 2 years and 18 age and sex matched healthy subjects. The delay period between fixation and target (“Go” or “No go”) visual stimulus was 2800-3000 ms. The early and late components of CNV - like slow negative waves (PMN1 and 2) have been studied in 1 sec pre-stimulus interval of delay period.

**Results:**

As compared to norm, the patients showed significantly increased latencies of saccades to correctly discriminated stimuli and higher percent of “errors saccades”. The amplitudes of No go-PMN1 and Go-PMN2 waves were also increased in patients. The amplitude foci of these waves were diffusely distributed in patients and mostly localized in frontal leads in norm.

**Conclusions:**

The findings assume some violation of anticipation for action (motor or inhibitory response) processes as well as an increase of presumably cortical activation during stimulus anticipation in the “Go/No go delay” saccadic paradigm in the early stage of schizophrenia.

**Disclosure:**

No significant relationships.

